# Harvesting information from Kaplan-Meier plots: part 1—detecting censoring

**DOI:** 10.1093/oncolo/oyae070

**Published:** 2024-06-06

**Authors:** Antonio Tito Fojo, Brian Labadie

**Affiliations:** Division of Hematology Oncology, Department of Medicine, Columbia University, New York, NY, United States; James J. Peters VAMC, New York, NY, United States; Division of Hematology Oncology, Department of Medicine, Columbia University, New York, NY, United States

## Abstract

This commentary focuses on the recent article by Sherry et al on missing elements in Kaplan-Meier plots, focusing not on what is left out, but what is often ignored—information that can often call the results, interpretation, and conclusion of a clinical trial into question.

We commend Sherry et al^1^ for their valuable submission on “missing visual elements” left out of Kaplan-Meier (KM) plots. In this brief primer, we focus not on what is left out, but more importantly, what is often *ignored*—information that can often call the results, interpretation, and conclusion of a clinical trial into question.

Figure 1B in the article by Sherry et al plots data generated “using a random number generator for a hypothetical randomized controlled trial comparing progression-free survival (PFS) assessed every 6 months following 1:1 randomization.” Interestingly, the KM they have generated has features consistent with that of a poorly conducted clinical trial. How so? PFS KM curves generated in properly conducted trials show “steps,” at least initially, representing progression occurring at the fixed assessment intervals. Beginning at 100% on the *y*-axis, the graph moves to the right as a flat line at 100% and only drops when the first assessment interval—in this case, 6 months—is reached. If patients had been neither censored nor assessed early (the latter a phenomenon often described as ascertainment bias), the magnitude of the drop precisely reflects the fraction of patients whose disease progressed at each assessment. For example, a drop to 86% if the tumors of 14 of the 100 patients enrolled had been scored as progression. Going forward, the line then remains flat at the level of 86% until the next assessment, at which time it would again drop by an amount reflecting the fraction of those with progression, and so on. However, even with nearly perfect conduct of a trial, the intervals of ascertainment gradually “spread out” as assessments are conducted either sooner or later than prescribed to accommodate a family vacation or other life events and eventually all KMs become continuums. In Figure 1B, we do not see steps, or even anything remotely resembling a step, an example of what one sees with heavily censored studies that also have ascertainment earlier than prescribed.

Let us begin with the problem of censoring and how we may estimate that. This exercise will also allow us to understand the problem of *informative* censoring, one that is increasingly confounding estimates of PFS, an important problem given PFS is increasingly accepted as a regulatory endpoint.

While KM curves can be used to estimate time-to-event outcomes in studies with incomplete follow-up (eg, in a KM for PFS, progression events or deaths that have not occurred by the data cutoff date), their validity is undermined when patients are censored due to reasons other than incomplete follow-up. With the first assessment interval in annotated Figure 1B as an example, censoring can occur in 2 ways: (a) anywhere between the start of treatment and the first restaging interval, when physicians or patients decide to discontinue an intolerable therapy with or without efficacy assessment, usually in those more ill or with worse disease biology—a concerning and informative type of censoring, or (b) at the time of the 6-month assessment after the first scan has been obtained and it shows an increase in tumor size but does not reach 120% of the starting quantity and thus not scorable as progression. In the latter case, the physician and patient who had been continuing treatment in the hope the therapy would help now realize it will not and decide to discontinue trial participation—an even more egregious example of informative censoring. In either of these 2 scenarios, censoring removes from one arm of a trial patients who would likely have progressed sooner. Why? Because those who are less fit are more likely to experience toxicity and disease progression. This is beneficial to the arm on which it occurs. How? Any time progression is scored one deducts the full penalty, a calculation that immediately reduces the progression-free fraction (the numerator) to the maximum amount, one patient. However, when censoring occurs, no immediate penalty is assessed but rather the denominator in the calculation for percent progression-free is reduced by one, because the patient is no longer enrolled in the study and the time of progression or death will never be known. Because the denominator (or patients still enrolled) is now one less, going forward each actual progression event will now be worth a bit more. But what has happened, is that the penalty of progression or death instead of being immediately deducted as a full patient progressing or dying has been “mortgaged” for the life of the study. For example, if 100 patients enroll and shortly thereafter 5 who are infirm, with possibly worse disease biology and the likelihood of progression are censored, the percent progression-free remains at 100%. If then in 10 patients, the disease progresses and only 85 remain on treatment, the percent progression-free is not 85% but rather 85/95 or 89.5%. Had the denominator remained at 100, each progression would have been worth 1%. But with the denominator now at 95, each progression “costs” 1.05% (10.5% drop in PFS as a result of 10 progressions), a beneficial trade-off given the imminent penalty for the 5 censored has been delayed.

It is thus important to be able to identify the number of patients censored and the time at which censoring has occurred. A rough idea can be had by looking at the “tick marks” along the KM curve. Tick marks represent the “censoring times,” but not the number censored; the tick marks are identical whether 1 or 4 or more patients were censored. “Thicker tick marks” represent censoring on successive time points. In studies with high censoring fractions [see studies of everolimus and cabozantinib as examples] the number censored exceeds the number of tick marks.

The easiest way to identify the *number* of patients censored is if the authors include the number in the KM plot. As seen in Figure 1B in the manuscript by Sherry et al, the total number of patients censored by each timepoint is found in parentheses below the *x*-axis. Unfortunately, with some exceptions including the *Lancet Journals* and *The Oncologist* going forward, the majority of journals, including those with the highest impact factors, do not require these numbers to be included in their KM PFS plots. But fortunately, the number of patients censored can be estimated quite accurately from the plot itself as shown in the annotated Figure 1B. To do this, one begins by drawing a line upward from 6 months on the *x*-axis. At the point where it crosses the curves, extend a line to the *y*-axis, then estimate at what percent it crosses the *y*-axis. Let us just look at the control arm in this example as if it were the experimental arm. PowerPoint estimates the length of the *y*-axis from 100% to 75% as 1.12 (“PowerPoint units,” or ppus) and the distance to where the red line intersects the *y*-axis as 0.28 ppus measuring down from 100%. With a distance of 1.12 ppus representing 25% on the *y*-axis, 0.28 ppus then equals 6.25%, and we estimate the fraction progression-free at 6 months in the control arm according to the study to be 100% minus 6.25%, or 93.75%. We know 2 numbers in the control arm: 173, the number enrolled, and 138, the number at risk going forward at 6 months, and we have estimated one number, 93.75%. Examining annotated Figure 1B, it appears from the tick marks and the slope of the curve that most of the patients who were censored were likely censored before the 6-month assessment. However, as discussed above, censoring can also occur at the time of the 6-month assessment. Because we do not know what fraction were censored before or after the time of the 6-month assessment, we estimate the number that would have been censored had all been censored in either way and take the average of these 2 estimates. We know 93.75% of those assessed had not progressed and we know that only 138 then continued on treatment, but we do not know how many were assessed to yield the 93.75%. In the first instance where all censoring occurred before and none after the assessment, the 138 “at risk” that continued treatment would have been 93.75% of 147 still on study and eligible for assessment [138 is 93.75% of 147], indicating 173-147 or 26 would have been censored before the assessment with 93.75% of the remaining 147 or 138 scored as progression-free and continuing on treatment, “at risk.” The second possibility is that none were censored before the assessment but that instead all were censored after having been assessed at 6 months (the figure tells us that is not the case, but we are calculating the extremes). Again, these are those that even though they had not scored as progression, felt benefit seemed unlikely to accrue and discontinued trial participation. Given the percent progression-free was 93.75%, and now assuming none were censored before assessment, then from the entire 173 assessed, 162 would have been progression-free [173 × 0.9375 = 162] with 24 then censored after assessment, leaving 138 to continue on treatment “at risk.” The actual number censored, 25, is the average of our estimates of 24 and 26 and suggests censoring occurred both before and after the 6-month assessment. As we look at the control curve and the number of tick marks, we conclude that most were censored before. We would note here 3 things:

(1) Censoring that is not informative is acceptable. This includes censoring because an individual has not been enrolled in a study long enough to reach a scheduled assessment. ln the overwhelming majority of trials, except those that report outcomes earlier than they should and that are still immature, all patients will have been enrolled in a study long enough to qualify for the first re-assessment and even the second. To confirm this, look for the day the last patient enrolled and the date of “data cutoff.” If this is longer than the time to first assessment or to any assessment of interest, then there should be no censoring before those times on the *x*-axis of the KM plot, because all such censoring would be considered potentially “informative.”
(2) If follow-up has been long enough, the process described for estimating the number censored in that first assessment interval can be repeated for the next assessment interval. In doing this, use the number at risk representing the number going forward as the starting number enrolled and the number you estimated as percent progression-free as the new 100%, with the subsequent estimated progression-free relative to this “new 100%.” In our example, 138 becomes the new 173 which was the number initially enrolled, and 93.75% becomes the new 100%. If the percent without progression at the next assessment is 80%, then divide 80 by 93.75 and find 85.3% as the percent progression-free in that interval, and use the number at risk moving forward from that next assessment in your calculations.
(3) Finally, when doing this with real and not “modeled data,” the spread of the numbers is larger than the 24-26 we calculated, but the average usually coincides well with the actual number censored. Also, a “short-cut” one can use, for example, in a meeting when a slide is presented, is estimate the 93.75% “visually,” multiply this by the number enrolled (173) and subtract the number “at risk” going forward (138), in effect, assume none were censored before assessment, and get a pretty good estimate of the number censored.

The foregoing discussion has referred to the more common occurrence, which is censoring of a more toxic experimental arm. In this case, informative censoring is beneficial to the toxic experimental arm by “mortgaging” across the duration of the trial, the penalty of what would have been almost certain progressions. But increasingly, we are seeing informative censoring in the control arm, often a standard of care treatment or a placebo abandoned by those who are more fit but who enrolled with the hope of randomizing to the experimental arm and, disappointed with their randomization, then discontinue participation to look for other options—note here that even blinded trials are often “pseudo-blinded”, in that, the patient or clinician can often determine to which arm randomization occurred [Ian Tannock, personal communication]. In this case, censoring is again informative but this time, it is *detrimental* to the arm in which it occurs (removing fit patients more likely to benefit) and when it occurs in a control arm benefits the experimental arm.

Informative censoring is often based on physicians doing “their best” for any one patient, which is why investigators need to think carefully before allowing bias to creep in and upset the equipoise that supported the trial design in the first place. If equipoise is lost, then an alternative trial design needs to be found. More on that in. the future.
